# Complete chloroplast genome sequence of *Bambusa pervariabilis* (Bambusodae)

**DOI:** 10.1080/23802359.2019.1710298

**Published:** 2020-01-24

**Authors:** Yang-Yang Zhang, Li-Li Fan, Jian-Lin Su, Lin-Zheng Xu, Min Li, Ling-Yan Chen, Jun-Dong Rong, Yu-Shan Zheng

**Affiliations:** a College of Forestry, Fujian Agriculture and Forestry University, Fuzhou, Fujian, China;; b Key Laboratory of Bamboo Institute, College of Forestry, Fuzhou, Fujian, China;; c College of Arts & Landscape Architecture, Fujian Agriculture and Forestry University, Fuzhou, Fujian, China

**Keywords:** *Bambusa pervariabilis*, plastid genome, phylogeny, Bambusodae

## Abstract

*Bambusa pervariabilis* is mostly produced in south China; usually cultivated on the banks of the rivers and near villages. We determined the complete chloroplast (cp) genome sequence of *B. pervariabilis* using Illumina sequencing data. The complete cp sequence is 139,393 bp, include large single-copy (LSC) region of 82,969 bp, small single-copy (SSC) region of 12,874 bp, a pair of invert repeats (IR) regions of 21,775 bp. Plastid genome contain 132 genes, 85 protein-coding genes, 39 tRNA genes, and 8 rRNA genes. Phylogenetic analysis based on 28 cp genomes indicates that *B. pervariabilis* is closely related to *Bambusa multiplex* in Bambusodae.

*Bambusa pervariabilis* is one of the important bamboo species in south China. It is also an irreplaceable forest vegetation revetment plant (Huang 2019). *Bambusa pervariabilis* is mainly distributed in Guangdong, Guangxi, Fujian province of China. The underground stem of *B. pervariabilis* belongs to axillary clump, the stem of which is lignified and has potential commercial value (Guo 2016). In this study, we report the complete chloroplast (cp) genome of *B. pervariabilis* based on Illumina pair-end sequencing data. Fresh leaf sample of *B. pervariabilis* was collected from Fujian province, China (Fujian Agriculture and Forestry University, Bamboo Garden, Fuzhou: 26°5′7′′N, 119°14′16′′E), and dried into silica gel immediately. The voucher specimen is kept at the Herbarium of College of Forestry, Fujian Agriculture and Forestry University (specimen code HTY013). DNA is extracted from fresh leaf tissue, with 500 bp randomly interrupted sequence by the Covaris ultrasonic breaker for library construction. The constructed library was sequenced PE150 by Illumina Hiseq Xten platform, with ∼2GB data generated. Illumina data were filtered by script in the cluster (default parameter: -L5, -p0.5, -N0.1). Complete plastid genome of *Arundinaria faberi* (GeneBank accession: JX513414) as reference and plastid genome of *B. pervariabilis* were assembled by GetOrganelle pipe-line (https://github.com/Kinggerm/GetOrganelle), it can get the plastid-like reads, and the reads were viewed and edited by Bandage (Wick et al. 2015). The cp genome annotation was assembled based on the comparison by Geneious v 11.1.5 (Biomatters Ltd., Auckland, New Zealand) (Kearse et al. 2012). The annotation result was drawn with the online tool OGDRAW (http://ogdraw.mpimp-golm.mpg.de/) (Lohse et al. 2013).

The complete plastid genome sequence of *B. pervariabilis* (GenBank accession:MN688610) was 139,393 bp in length, with a large single-copy (LSC) region of 82,969 bp, a small single-copy (SSC) region of 12,874 bp, and a pair of inverted repeat (IR) regions of 21,775 bp. The complete chloroplastid genome contained 132 genes, including 85 protein-coding genes, 39 tRNA genes, and 8 rRNA genes. The complete genome GC content was 44.2%. In order to reveal the phylogenetic position of *B. pervariabilis* with other members of Bambusodae, a phylogenetic analysis was performed based on 26 complete cp genomes of Bambusodae, and two taxa (*Acidosasa purpurea*, *Ampelocalamus actinotrichus*) as outgroups. All of them were downloaded from NCBI GenBank. The sequences were aligned by MAFFT v7.307 (Katoh and Standley 2013), and the phylogenetic tree constructed by RAxML (Stamatakis 2014). The phylogenetic tree showed that *B. pervariabilis* was most closely related to *Bambusa multiplex* with strong support (Figure 1).

**Figure 1.**
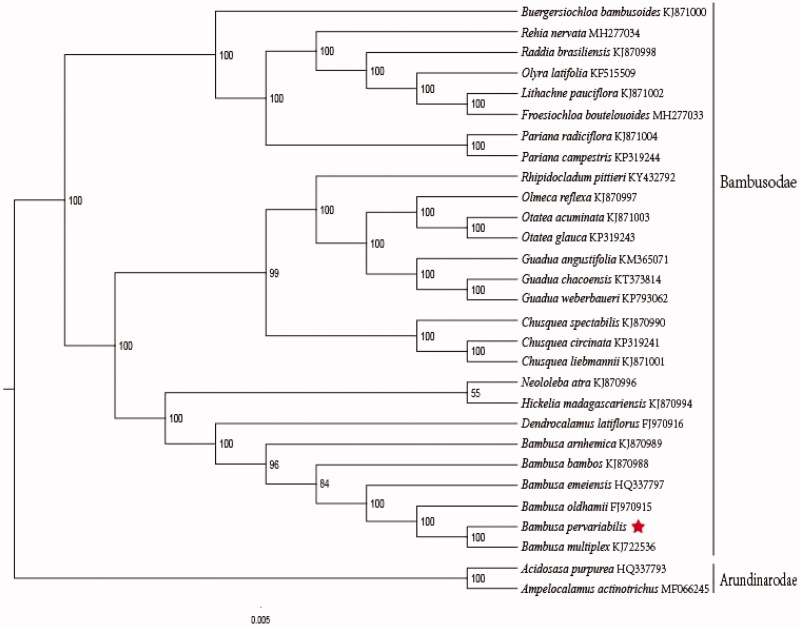
Phylogenetic analysis of 26 species of Bambusodae and two taxa (*Acidosasa purpurea*, *Ampelocalamus actinotrichus*) as outgroup based on plastid genome sequences by RAxML, bootstrap support value near the branch.
